# Straightforward Procedure for Laboratory Production of DNA Ladder

**DOI:** 10.1155/2012/254630

**Published:** 2012-02-12

**Authors:** Vo Thi Thuong Lan, Pham Thi Thanh Loan, Pham Anh Thuy Duong, Le Thi Thanh, Ngo Thi Ha, Ta Bich Thuan

**Affiliations:** Faculty of Biology, Hanoi University of Science, Vietnam National University, 334 Nguyen Trai Street, Thanh Xuan, Hanoi, Vietnam

## Abstract

DNA ladder is commonly used to determine the size of DNA fragments by electrophoresis in routine molecular biology laboratories. In this study, we report a new procedure to prepare a DNA ladder that consists of 10 fragments from 100 to 1000 bp. This protocol is a combination of routinely employed methods: cloning, PCR, and partial digestion with restriction enzymes. DNA fragments of 100 bp with unique restriction site at both ends were self-ligated to create a tandem repeat. Once being cloned, the tandem repeat was rapidly amplified by PCR and partially digested by restriction enzymes to produce a ladder containing multimers of the repeated DNA fragments. Our procedure for production of DNA ladder could be simple, time saving, and inexpensive in comparison with current ones widely used in most laboratories.

## 1. Introduction

A DNA ladder is a solution of DNA molecules of different lengths used in agarose or acrylamide gel electrophoresis. It is applied as a reference to estimate the size of unknown DNA molecules that were separated based on their mobility in an electrical field through the gel. Thus, DNA ladders are essential molecules routinely used in every DNA dealing laboratory. Digestion with restriction enzymes of known-length DNA fragments from natural sources such as lambda [1], simian virus 40 [2], and plasmid [3] creates the ladder fragments the lengths of which are dependent on restriction enzyme sites thus, not fully controlled. To overcome this disadvantage and to make DNA ladders more flexible, DNA engineering was developed [4]. Perhaps, for commercial purpose, DNA manipulation for producing DNA ladder fragments became confidential. Typically, a DNA fragment that contains a tandem repeat units separated by the same unique restriction enzyme sites was cloned into a plasmid and then partially digested to produce a ladder with multimers of the repeats [5, 6]. However, the more clear visibility of small size fragments requires the higher amount of plasmid used in cleavage reaction. Recently, many laboratory protocols describing the preparation of DNA ladders by employing the polymerase chain reaction (PCR) method have been reported [7–9]. This method involves either the simultaneous amplification of a DNA target using primer sets [8] or the separate amplification of a different DNA targets using specific primers [9, 10]. However, using simultaneous primer sets is often difficult to be reproductive because of problematic optimization of PCR conditions, while using separate primer set for a particular fragment of ladders causes a laborious task.

Here, we describe a method to produce 100 bp DNA ladder, which minimizes experimental disadvantages mentioned above. Based on our protocol, any laboratory can make its own 100 bp DNA ladder instead of purchasing from commercial sources.

## 2. Materials and Methods

### 2.1. Production of 100 bp Sequence

A DNA fragment of length 100 bp was amplified from a known DNA sequence by using two specific primer sequences containing a site of recognition enzyme at the 5′ ends. Since any known sequence could be chosen, PCR conditions were optimized depending on appropriate DNA template and designed primers. In our experiment, the specific primers contained a site of* Sma*I; thus, they were named *Sma*-100F and *Sma*-100R, respectively.

### 2.2. Production of Multimer of 100 bp Repeat

The 100 bp PCR product was digested completely by the *Sma*I enzyme and purified by using QIAquick PCR Purification Kit and DNA was eluted by 50 *μ*L of water as recommended by manufactory (QIAGEN). One hundred ng of *Sma*I-treated DNA was subsequently self-ligated in the reaction of 16 *μ*L from which 1 *μ*L was used as a template for PCR with the two primers* Sma*-100F/R. Since self-ligated DNA templates were mixture of one to multimers of 100 bp repeats, so PCR product was visible as a smear on 1.5% agarose gel after electrophoresis. The smeared DNA ranging from 500 bp to 1000 bp was isolated and cleaned using gel elution kit (QIAquick Gel Extraction-QIAGEN) and cloned into pGEM-T vector system (Promega, Madison, WI, USA). A clone containing an insert of 800 bp in length was selected, sequenced, and named pGEM-800.

### 2.3. Amplification of Multimers of 100 bp

**Figure 1:**
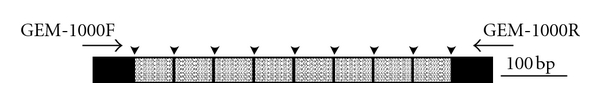
Schematic structure of pGEM-800 plasmid. The plasmid contains an insert of 800 bp which is a multimer of 100 bp repeats separated by the same unique *Sma*I restriction enzyme site (indicated by arrows). The GEM-1000F and GEM-1000R primers were designed to flank 800 bp insert plus 200 bp in length based on the pGEM sequence.

Based on the pGEM-T sequences flanking the insert of 800 bp, two specific primers, named GEM-1000F and GEM-1000R, were designed in order to amplify a DNA fragment of 1000 bp in length using pGEM-800 as the template (Figure 1). These two specific primers were used: GEM-1000F 5′-ttg taa aac gac ggc cag tga att gta at-3′ and GEM-1000R 5′-cta ttt agg tga cac tat aga ata ctc aag-3′. PCR conditions were as follows: 20 *μ*L of 5 × PCR buffer (GoTaq-Promega), 5 *μ*L of dNTPs (2.5 mM), 10 *μ*L of each primer (2.5 nmoles/*μ*L), 1 *μ*L of template DNA (50 ng/*μ*L), and 2.5 U of GoTaq DNA polymerase in a final volume of 100 *μ*L. The thermal cycling profile was 94°C for 15 s, 65°C for 30 s, and 72°C for 1 minute. Amplification was carried out for 40 cycles in a Gene-AmpPCR System 9700 thermocycler (Applied Biosystems, FosterCity, CA, USA). The PCR product was 1000 bp like the sum of 800 bp DNA insert plus 2 flanking fragments. Afterward, this DNA fragment was partially digested by *Sma*I to yield all the segments of 100 bp DNA marker ladder. The *Sma*I digestion reaction was as follows: 1 *μ*g DNA of 1000 bp in length was mixed with 2 *μ*L of *Sma*I (10 U/*μ*L) and incubated at 30°C for 5 minutes, then enzyme inactivation was carried out at 65°C for 10 minutes. The digested DNA was concentrated by ethanol precipitation and dissolved in 200 *μ*L of TE8 (10 mM Tris-HCl, 1 mM EDTA, pH 8). Finally, 5 *μ*L of the 100 bp DNA ladder was subjected to DNA electrophoresis on 2% agarose or 12% acrylamide gels containing ethidium bromide. An image was obtained by ChemiDOC XRS (BioRad).

## 3. Results and Discussion

**Figure 2:**
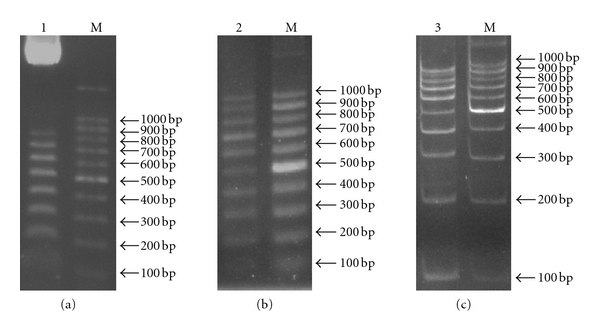
Production of 100 bp DNA ladder. (a) The pGEM-800 plasmid was partially digested by *Sma*I enzyme producing 8 fragments in length of 100–800 bp. (b, c) Electrophoresis of the prepared 100 bp DNA ladder. A 5 *μ*L (lane 2) or 3 *μ*L (lane 3) of DNA ladder prepared in the present study and 100 bp DNA marker from Takara (M) were submitted to 2% agarose (b) or 12% acrylamide (c) gel electrophoresis, respectively.

Our study successfully produced 100 bp DNA ladder with 10 fragments ranging from 100 to 1000 bp. Our procedure contained three steps. In detail, the first step was to make a 100 bp DNA fragment from a known sequence by using two specific primers that contain restriction site at the 5′ ends. One fragment out of sequenced ones in our experiments that contains *Sma*I recognition site at the 5′ end was cloned; thus, we did make the specific primers *Sma*-100F and *Sma*-100R. The selection of sequences and specific primers for preparation of 100 bp DNA fragment is easy and flexible in most laboratories working on DNA. The second step was cloning PCR products which were reamplified from self-ligated DNA of *Sma*I-digested 100 bp fragments. Using universal primers of pGEM-T vector, the largest insert of 800 bp was quickly selected, and recombinant plasmid containing this fragment was named pGEM-800. It should be noted that some inserts larger than 800 bp could be selected when the self-ligated reaction was performed by commercial DNA ligation kits with a special efficiency in DNA ligation. Thus, a 100 bp DNA ladder with a range outside of 1000 bp could be generated. Since the same unique *Sma*I restriction site lies at each junction of the 100 bp repeat units, the pGEM-800 could be partially digested by this enzyme to produce a 100 bp DNA ladder (Figure 2(a)). In this case, our DNA ladder contained 8 fragments ranging from 100 to 800 bp. We observed that a large amount of pGEM-800 plasmid is required for clear visibility of small fragments as 100 bp–200 bp. In addition, partial digestion of plasmids was dependent on plasmid conformation (supercoilled, circular and linear forms); thus, it was not easy to reproduce.

**Figure 3:**
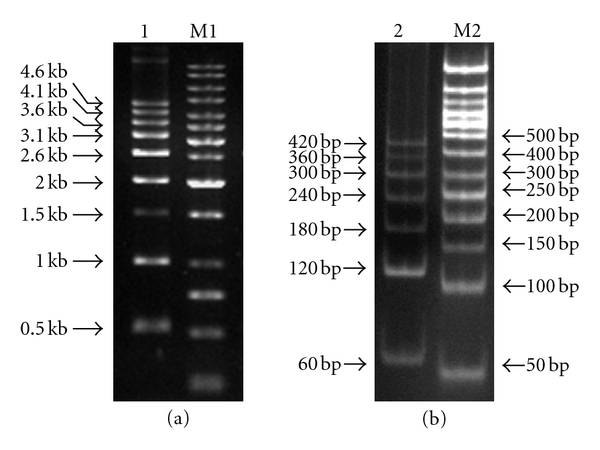
(a) 500 bp DNA ladder on 1% agarose gel (lane 1) and (b) 60 bp DNA ladder on 12% acrylamide gel (lane 2) were prepared on the basis of strategy presented in this study. M1: 1 kb DNA marker (NEB). M2: 50 bp DNA marker (Fermentas).

In order to overcome the disadvantages mentioned above, our protocol was used in third step during which a PCR product of 1000 bp was amplified from pGEM-800 using two primers GEM-1000F and GEM-1000R. Both primers were designed on the basis of pGEM-T sequences which are located at 100 bp far from the upstream and downstream of cloning site. Therefore, the size of 1000 bp of PCR product was the sum of the 800 bp insert plus 200 bp. A huge amount of linear formed DNA was quickly made by PCR reaction, providing adequate materials for reproduction of DNA ladder. In fact, from 1 mL of PCR product we can produce 300 runs of DNA 100 bp ladder for electrophoresis on agarose gels or 600 runs on 12% acrylamide gels (Figure 2). Clearly, this strategy was quite straightforward, time saving, and especially inexpensive. For instance, the price of 100 bp DNA ladder for 50 runs is listed 110 USD (G2101-Promega) and 53 USD (N3231S-New England Biolabs) on available web sites. We estimated that our protocol spends only 3 USD for materials to produce 50 runs of 100 bp DNA ladder. In addition, our strategy is flexible for producing different kinds of DNA ladders. For instance, labeled 100 bp DNA ladder can be produced by using labeled dNTPs in the PCR amplification. In fact, we have successfully produced the 60 bp DNA ladder and the 500 bp DNA ladder supplied for our specific needs (Figure 3). We estimated that it takes approximately 2 weeks for laboratory works to carry out this procedure. Once self-ligated insert with desirable length was cloned, it took a few hours to produce a huge amount of DNA ladder. Additionally, size range of each ladder could be broadened by repeating this protocol using PCR product amplified from recombinant plasmid. For instance, the DNA fragment of 1000 bp was self-ligated, cloned into a plasmid and DNA fragments with favorable size could be selected. A minor disadvantage in using the self- and quickly produced DNA ladders is unknown amount of DNA in each band. The commercial DNA ladders compensate this demand even though this information is not always needed.

Compared with conventional methods for producing DNA ladders, our strategy reported in this paper is simple and flexible for preparation of the 100 bp DNA ladder that contains 10 fragments ranged from 100 to 1000 bp. Our strategy could be applied for producing different kinds of DNA ladders of good quality and could be useful for most laboratories.
